# Systematic Study of the Nanostructures of Exfoliated Polymer Nanocomposites

**DOI:** 10.1021/acs.macromol.3c00575

**Published:** 2023-09-14

**Authors:** Suellen Pereira Espíndola, Jure Zlopasa, Stephen J. Picken

**Affiliations:** † Advanced Soft Matter, Department of Chemical Engineering, Faculty of Applied Sciences, Delft University of Technology, Van der Maasweg 9, 2629 HZ Delft, The Netherlands; ‡ Environmental Biotechnology, Department of Biotechnology, Faculty of Applied Sciences, Delft University of Technology, Van der Maasweg 9, 2629 HZ Delft, The Netherlands

## Abstract

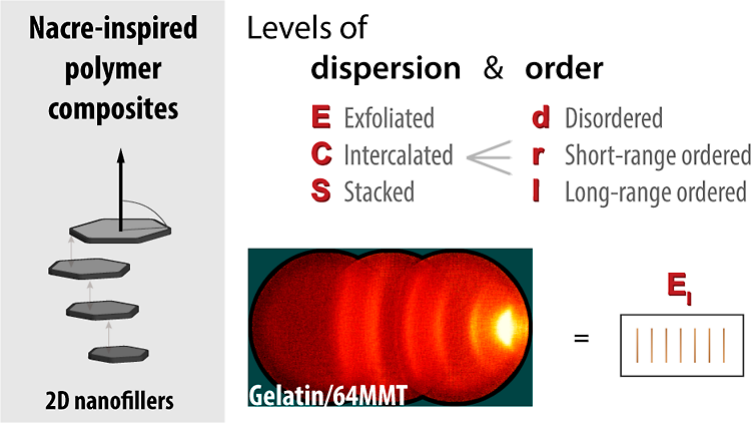

High-performance bioinspired materials have shown rapid development over the last decade. Examples are brick-and-mortar hierarchical structures, which are often achieved via solvent evaporation. Although good properties are claimed, most systems are composed of stacked or intercalated platelets. Exfoliation is a crucial step to give ultimate anisotropic properties, *e.g.*, thermal, mechanical, and barrier properties. We propose a general framework for all the various types of micro-scale structures that should be distinguished for 2D filler nanocomposites. In particular, the exfoliated state is systematically explored by the immobilization of montmorillonite platelets via (gelatin) hydrogelation. Scattering techniques were used to evaluate this strategy at the level of the particle dispersion and the regularity of spatial arrangement. The gelatin/montmorillonite exfoliated nanostructures are fully controlled by the filler volume fraction since the observed gallery *d*-spacings perfectly fall onto the predicted values. Surprisingly, X-ray analysis also revealed short- and quasi long-range arrangement of the montmorillonite clay at high loading.

## 1 Introduction

Natural materials are intriguing as they can display complex, highly regular nano-to macro-architectures, which are fabricated under ambient conditions.^1^ A common example is the “brick-and-mortar” micro-structure of nacre, which inspired the design of polymer composites for the past decades.^2^ In essence, the hybrid nacre-like materials consist of highly ordered inorganic platelets which are bound in a lamellar manner. Several mimetic and bioinspired materials have been constructed either from suspension/melt mixing (top-down) or in situ techniques (bottom-up).^2−4^ The design of hierarchically structured composites from 2D nanoparticles has already been extended to clay silicates, (reduced) graphene oxide, boron nitride, MXenes, dichalcogenides, among others.^5−8^ In general, the anisotropic materials show desired thermal, mechanical, conductive, and barrier properties. For instance, they combine excellent stiffness and toughness.^4,9,10^ Nevertheless, in order to achieve tailored structural properties, the right choice of building blocks and fabrication strategy become important.

Processing conditions can dramatically influence the obtained hierarchical structure. Several processes have been reported, such as suspension casting, doctor blading, vacuum-assisted self-assembly, melt compounding, in situ polymerization, etc.^2,11^ About a decade ago, bioinspired polymer nanocomposites saw much advance with water-based and large-scale methodologies, akin to paper-making.^9,10,12,13^ Since then, the focus has shifted to the optimization of polymeric core–shell particles, followed by solvent removal.^5,6,14^ The ideal conditions to realize aligned assemblies have been extensively adjusted. Nevertheless, these often are formed by intercalated structures, with restacking still observed at higher filler concentrations.^2,15−20^ Above all, these efforts have led to a great number of mechanisms being proposed for polymer-particle association and (re)organization.

The main drive behind new technologies is the same: improving the measured anisotropic properties over the in-plane arrangement. As a strategy, the logical approach should be to keep the high aspect ratio of 2D filler (exfoliated state) and, if feasible, aim for high loadings. This would ensure the nanoscale properties, and functionalities, to be translated as much as possible to the bulk material. However, as previously discussed, composites do not frequently achieve the formation of exfoliated nanostructures, with limited polymer insertion in between the particles, although an exception might be made for samples from layer-by-layer or multilayer deposition approaches.^21,22^ Furthermore, many applied studies exclusively investigate whether there is polymer intercalation onto the integral layers of the 2D filler, which is accompanied by a synergetic improvement of properties. High filler contents, i.e., above 20 wt %, are equally unexplored. Thus, the quality of (nano)dispersion needs more systematic evaluation, guiding the way to better methods and ultimate properties from all exfoliated samples.

**Chart 1:**
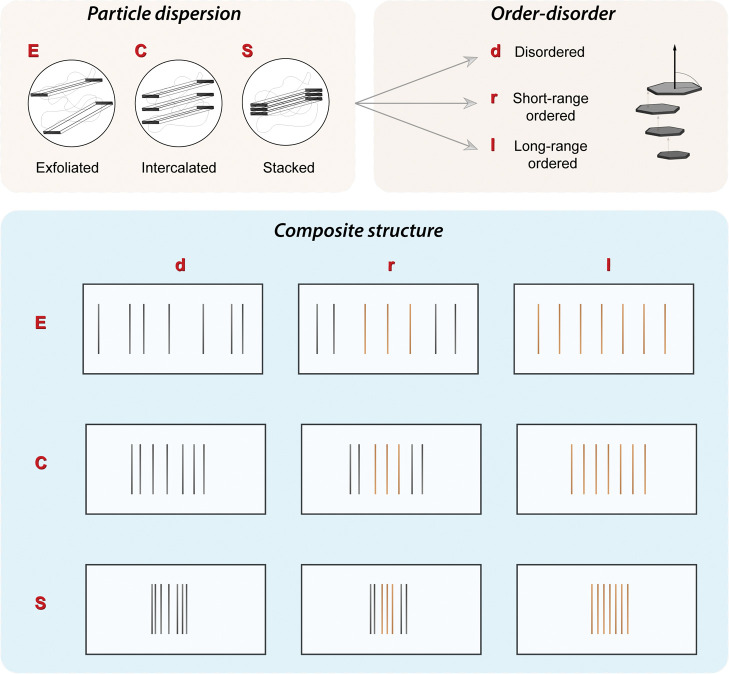
Structure Classification of Polymer Nanocomposites Based on Level of 2D-Filler Dispersion and Positional Order–Disordera

Finally, there is still lack of consensus about the classification of composite micro-structures, where the difference between intercalated and exfoliated states remains elusive. For instance, highly interlocked systems can also show a certain level of regularity due to the particles’ flat geometry. In other words, there can be a positional order–disorder transition with the filler concentration. So far, we have not seen a complete overview of the combined effects of the level of 2D material dispersion and associated positional order to the (nano)structure. In Chart 1, we illustrate how this coupling (dispersion and regularity) will lead to many more composite nano-to meso-phases. The schematic cross-sectional structures are representative of the mean 2D-material organization in a frame. This distribution is the only information required for the nanocomposite structural classification. If desired, the superstructure could be further elucidated to a three-dimensional organization with the aid of electron microscopy coupled to ion-milling^23^ or X-ray tomography of the composite. The orientational order of the dispersed phase is for now ignored, even though alignment will clearly have an influence on the anisotropic properties. Thus, we introduce a general framework on all the possible phases of dispersion and positional order present in polymer/platelet composites.

a The length of 2D materials is typically between 100 and 5000 nm and exfoliated thickness around 1–10 nm.

### 1.1 Nanostructure Classification Based on 2D Particle Dispersion and Positional Order

#### 1.1.1 Particle Dispersion

Depending on the level of initial dispersion and system compatibility, 2D materials (platelets or sheets) can yield exfoliated (*E*), intercalated (*C*), or stacked (*S*) composite structures. Restacking into aggregates, or tactoids, is one of the most frequently addressed issues in nanocomposite preparation and processing.^24−27^ When platelets are stacked (*S*) they are phase separated from the polymer matrix, indicating chains do not diffuse in between the individual layers, which remain immiscible. In X-ray diffraction (XRD), the gallery *d*-spacing, *d*_001_, corresponds to the initial bulk material. In the specific case of sheets, additional phenomena such as wrinkling and fold-overs also come into play.^28^ Further on, when the 2D filler is considered intercalated (*C*), there is still aggregation, but an increase in the *d*-spacing between integral layers is observed. This is attributed to polymer diffusion or expansion of the gallery. The distance between 2D units is enlarged and, most importantly, fixed. In the case of exfoliation (*E*), sometimes also called delamination, there is not a fixed length between the single layers but an average spacing value. This also means that the net interparticle force between the individual colloids is repulsive. A point often overlooked is that the truly dispersed state, *E*, only happens when there is homogeneous distribution of the 2D material in the polymer matrix. Therefore, the mean spacing of 2D units must be dictated by the total volume of separated particles. In this context, a criterion for exfoliation based on some required distance is not appropriate as the distance depends on the filler volume fraction.

#### 1.1.2 Positional Order–Disorder

In addition to the dispersion, nacre-like composites with high aspect ratio particles can also develop a degree of periodicity. Considering samples from suspension or melt mixing, this is rare in contrast to the commonly attainable orientational order of anisotropic particles. Nevertheless, regularity can happen in a few systems, especially if there are self-assembling molecules and particularly toward higher 2D filler fractions. When a structure is disordered (*d*), there is no regular correlation between the position of individual 2D particles, however delaminated or within a stack. Short-range ordered (*r*) systems will show some localized structural positioning. The extent of periodicity is determined by a correlation length, calculated from fitting a Lorentz-type function over scattering profile. This length is distinct from the platelet gallery spacing (dispersion). For the special cases of (quasi) long-range order (*l*), the materials’ structure will resemble that of a 1D crystal. In practice, these long-range ordered structures will have a finite size or have a finite correlation length domain length, representing the extent of periodicity of a pile of regularly spaced platelets. The finite domain length is commonly fitted to a Gaussian distribution function. In XRD, short- and (quasi) long-range order can also be studied from the shape of the peak width of multiple {00*l*} reflections in the scattered intensity profile.^29^ The more narrow these peaks are, the higher is the probability of finding a scatterer at a position that matches that of a crystal lattice plane. Regardless of the degree of order in the system (*d*, *r*, and *l*), it is imperative to notice that this distinction is relevant irrespective of the dispersion quality (*E*, *C*, and *S*).

In the field of high-performance nacreous materials, the mechanisms to actively achieve nanoparticle exfoliation and hierarchical structure features are still missing. Therefore, the focus herein is on a strategy to fabricate exfoliated nanostructures, even at high loading, by preventing reaggregation. We hypothesize that an earlier immobilization of platelets would enable us to preserve, initially, the exfoliated system. To test this, we have investigated a water-based system using montmorillonite clay (MMT), up to a high fraction of 80 wt % on composite basis, in a thermo-reversible gelatin network. The fast-gelling reaction should immobilize the platelets early enough to ensure less or no virtual stacking during solvent evaporation. Therefore, a network formation that prevents filler relaxation would result in exfoliated structures and keep the MMT high aspect ratio. The study is a systematic investigation of the evolving structures from exfoliated platelets locked in a hydrogel matrix. The underlying goals are two-fold: to reiterate the importance of achieving exfoliated nanostructures (level of dispersion) in the design of functional, nacre-like composites; and to clarify the nomenclature of 2D material organization. We refrain from addressing particle alignment here since this topic shall be discussed more extensively in a follow-up communication.

## 2 Results and Discussion

The gelatin A/MMT bionanocomposites were prepared by solvent casting 3% solid suspensions at RT (method in Supporting Information Text S1). The formation of an unfragmented hard gel network, like in pristine gelatin, was visually observed in hybrids up to a 20 wt % MMT content (Figure S4). The final filler mass was determined by thermogravimetric analysis (TGA, Supporting Information Text S2) and converted to a composite volume fraction percentage *X*, herein denoted as *X*MMT. Curiously, the obtained films showed high translucency up to sample 11MMT, while volume fractions above 33–64% had increasing haziness (Figure S5). Because the aligned nanostructures are less scattering the visible light, transparency is often taken as an identifying feature of a homogeneous and well-dispersed phase. It is true that transparency can indicate the absence of 2D filler aggregation^30,31^ but at times homogeneous systems, for instance locally crystalline (spherulites in semicrystalline polymers) or anisotropic arrangements (nematic fluids), can show opacity.

**Figure 1:**
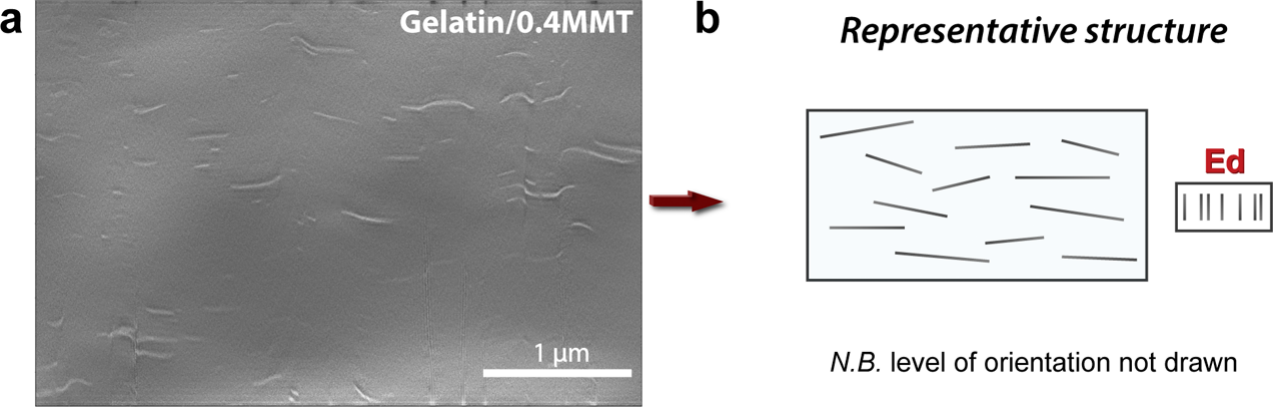
FIB-SEM cross-sectional image of gelatin/0.4MMT composite (a) and its representative nanostructure (b), classified as exfoliated disordered (Ed).

We further examined the gelatin/MMT samples with focused ion beam scattering electron microscopy (FIB-SEM). Because of the large difference between soft/hard phases, the ion milling step was slightly uneven in the vertical direction, and image treatment was necessary (Supporting Information Text S3). The obtained micrographs allow for inspecting the morphology of film cross-section. All the bionanocomposite samples showed the expected in-plane orientation (Figures S2 and S3). No large-scale aggregation or restacking was observed up to 64MMT. In Figure 1, we show a very diluted sample, containing only 0.4% MMT in volume. At this unique regime, we could see MMT platelets separately and measure their lateral dimension, of roughly 130–270 nm. To investigate the 2D material thickness, electron scattering techniques should not be relied upon due to charge density and contrast blurring effects. However, they can provide for a qualitative estimation of particle width, degree of separation, and extent of exfoliation.^32^ The 0.4MMT sample can be classified as exfoliated disordered (Ed) because homogeneous spreading of nanosized particles is observed but without any observable periodicity. Only a global evaluation the particle dispersion could be carried out for higher platelet fractions, 11MMT and 64MMT, because charging effects hindered discrete particle visualization (Figure S2). In general, we note the absence of clay aggregates based on uniform scattering. For these samples, exfoliated or intercalated phases are probable, considering the uniform distribution of densely layered structures.

**Figure 2:**
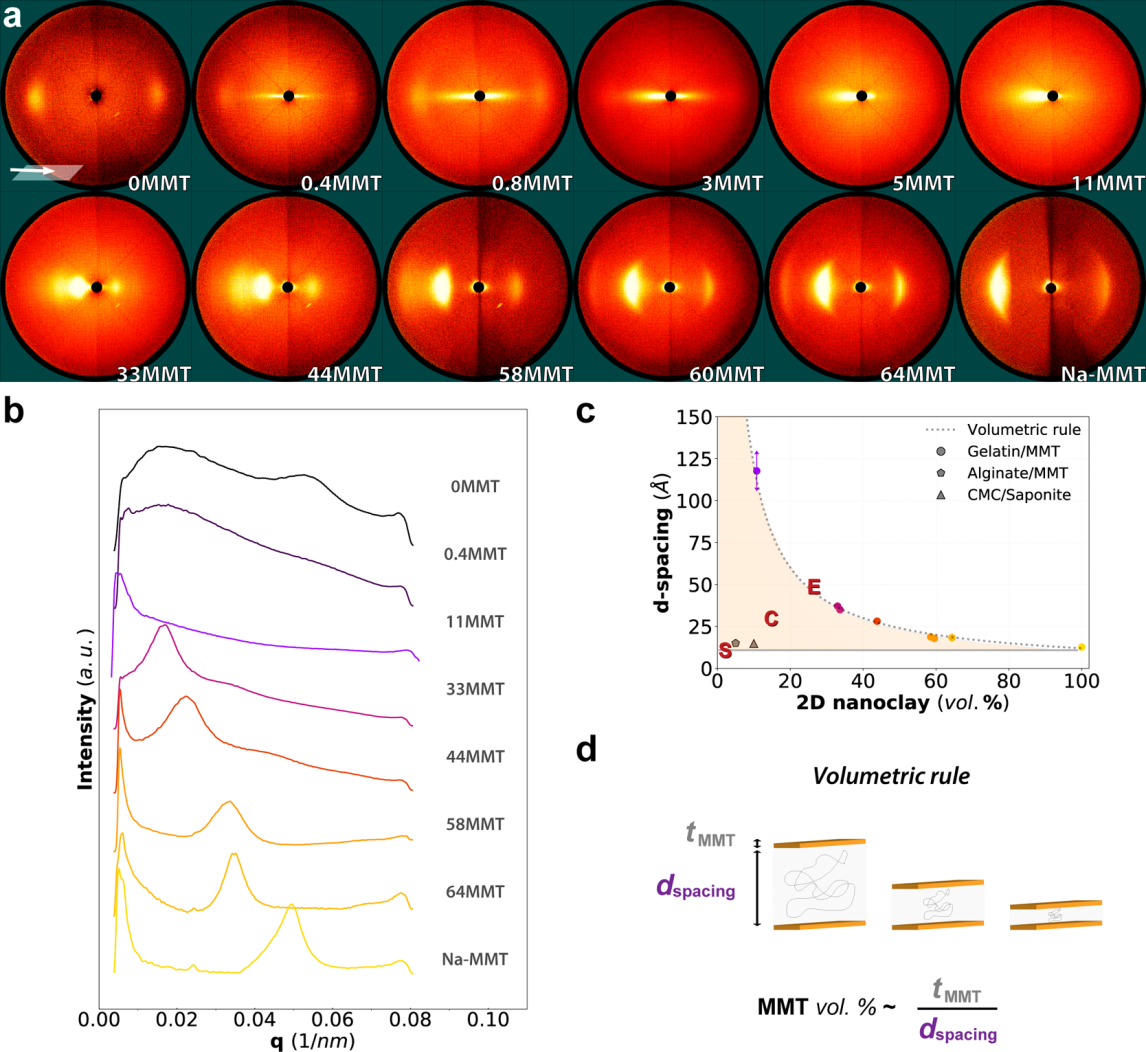
(a) 2D transmission X-ray scattering images of gelatin/MMT samples with varying volumetric MMT composite contents at beam inclination parallel to the plane of the films (90.5–94° range of glancing angle). The scattering patterns reveal anisotropy and a progressive change in basal reflections with increasing clay loadings. (b) Wide-angle X-ray diffractograms depict a peak shift of *d*_001_ MMT reflections toward higher angles starting from sample 11MMT. (c) Comparison between experimental and calculated *d*-spacings derived from the *d*_001_ MMT reflection. The thickness of a delaminated Na-MMT platelet was obtained by reciprocal fit of *d*_001_ lengths and extrapolating to origin (virtual zero concentration). Alginate and carboxymethyl cellulose composite data, respectively, from Zlopasa et al.^30^ and Ebina and Mizukami.^18^ (d) Illustration depicting the theoretical basis for calculating *d*-spacing, where a reciprocal volumetric rule of concentration of MMT is assumed; *t*_MMT_ is the thickness of a single platelet, and *d*_spacing_ is the platelet interspace.

Wide angle X-ray scattering (WAXS) was used to investigate the basal spacing of MMT (Figure 2). The patterns were obtained with the incident beam at a glancing angle parallel to the film surface, as shown in Figure 2a. The angle applied was iterated to ensure that the scattering vector properly intersects the Ewald sphere. This transmission mode is very sensitive to the local 2D material arrangement and the rather high level of in-plane orientation. We could observe distinct trends between sample groups 0.4 to 11MMT and 33 to 64MMT. A clear streak was observed up to 5MMT, while for 11MMT, an ill-defined wedge shape appeared. At higher filler volume fractions, from 33MMT, there were clear equatorial arcs, typical of anisotropic MMT layers. The first detection of a basal 001 reflection was at 11MMT, corresponding to an average spacing of *d*_001_ = 120 Å. Remarkably, a gradual change in the peak position of clay reflection was observed with the increasing clay content (Figure 2b). With this, the corresponding *d*-spacings decreased down to 18 Å. These distances are larger than the back calculated pure MMT basal spacing of 12 Å (Figure 2c), which confirms that there was gelatin intercalation into the clay galleries for all samples. Additionally, the orientation seen in the pristine Na-MMT film likely developed from flocculation (edge-face attraction), yielding an arrangement like in a random pile of cards.^33^

In a defined two-dimensional space, if we consider perfectly aligned 2D nanoparticles that are not allowed to interact, the volume fraction can be defined by the summed thickness of all particles over the determined space length. By analogy, the concentration of particles should be proportional to the thickness of particles over the interparticle spacing (Figure 2d). For this reason, the apparent *d*-spacing is inversely proportional to the MMT volumetric content, decreasing with the filler concentration. Within the realm of colloid science, the progression of the mean interparticle separation in correlation with the volume fraction is named the swelling (or dilution) law.^34,35^ This volumetric rule is what truly defines an exfoliated structure and is valid to any form of 2D platelet/sheet nanocomposite. Therefore, all the studied concentrations for gelatin/MMT seemed to follow the exfoliated regime. From 11MMT up to 64MMT, there is an excellent agreement between experimental and theoretical exfoliated *d*-spacings derived from the integrated 001 reflections (*R*^2^ = 0.993). Despite unusually high clay fractions, there is virtually no re-stacking and phase separation over a wide concentration range (up to 64 vol %). This is encouraging because examples of perfect exfoliation are limited, with often high temperature and pressure required.^27^ In comparison, other successful 2D silicate bionanocomposites have been reported, usually at lower loadings, and, even so, fall inside the intercalated regime (Figure 2c).^18,30^

**Figure 3:**
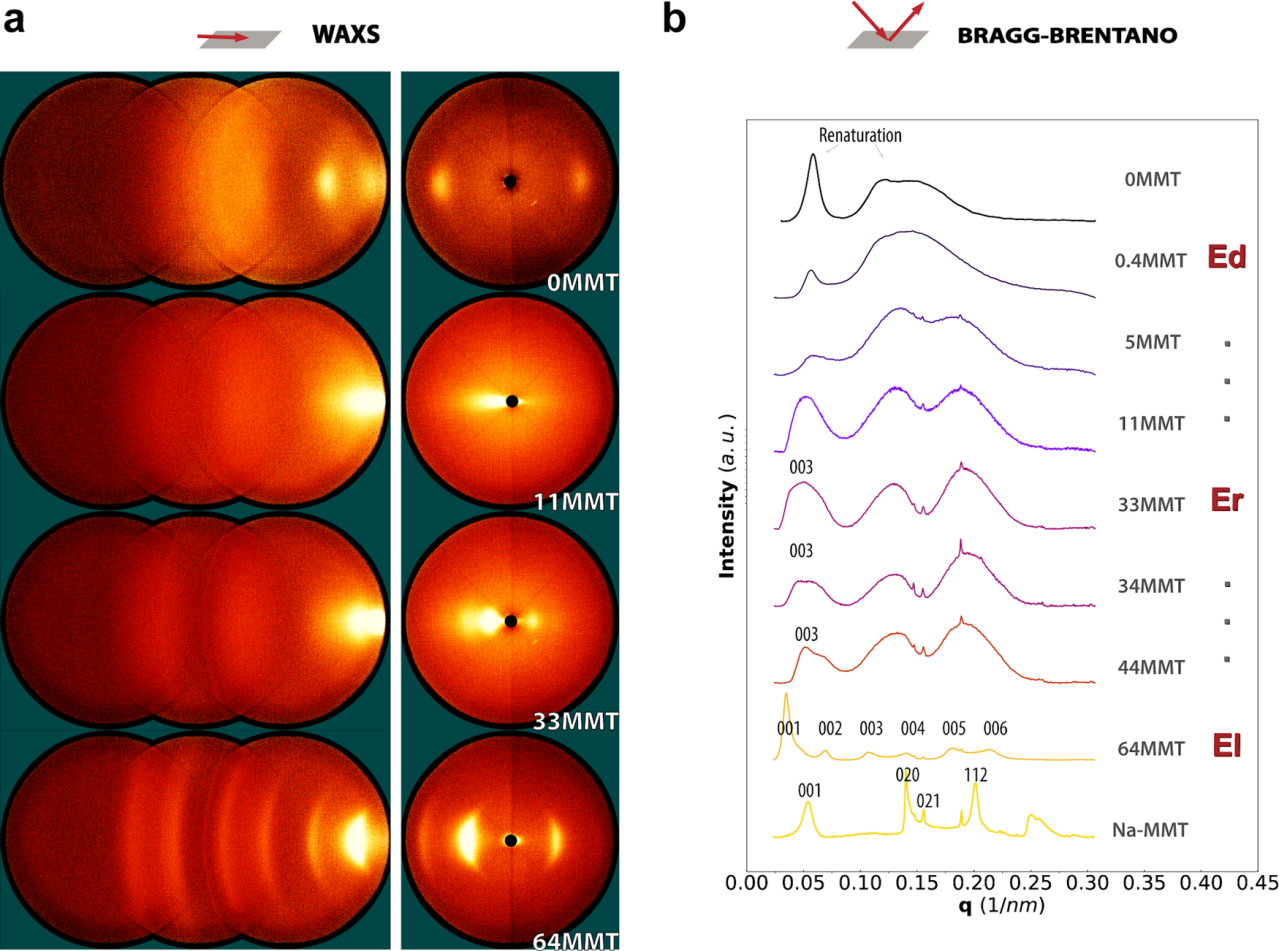
(a) 2D transmission X-ray scattering images at higher scattering vectors *q* of gelatin/MMT samples with varying volumetric MMT composite contents at beam inclination parallel to the plane of the films (90.5–94° range of glancing angle). (b) Bragg–Brentano X-ray diffractograms on films show a decrease in gelatin renaturation and appearance of multiple MMT reflections with increasing loading fractions. Multiple reflections are key to identify regularity (positional order).

The WAXS diffraction patterns were also obtained at higher *q* scattering vectors by moving the detection setup (Figure 3a). Interestingly, for neat gelatin, the scan showed structural arrangement that is derived from the crystallization of collagen-like helices (*q* 0.05 nm^–1^) and a broad peak related to the peptide bonds.^36^ The WAXS patterns showed that samples 33MMT and higher have additional reflection peaks (also broad [002] and [003] features), corresponding to higher orders of the MMT lamellar planes (Figure S7). Surprisingly, the sample 64MMT showed many additional reflections, all the way up to [006]. To investigate this further, the layered bionanocomposites could also be studied in X-ray analysis in Bragg–Brentano geometry, allowing for higher intensity levels (Figure 3b). The 1D diffractograms are used to better detect the presence of higher order clay reflections [00*l*]. Additional lattice reflections ([002] and [003]) were confirmed for samples 33MMT and higher around *q* range 0.05–0.20 nm^–1^, which can be interpreted as evidence of improved layer regularity. The sample 64MMT showed remarkable quasi long-range order and six reflection peaks. However, due to polydispersity in the particle’s width, this should not be confused with a real one-dimensional lattice. The periodic systems were modeled by a Gaussian distribution function over *q*. The correlation length or (para)crystal domain size were calculated via the Scherrer equation^37^ for both 001 and 003 reflections, which were observed using different scattering geometries (Table 1). In the case of gelatin/MMT composites, the length *L* translates into the relative extent of short- or long-range positional order. We note that the platelet arrangements might be paracrystalline since ξ seemed to depend on the X-ray reflection (Supporting Information Text S4). For 64MMT, a substantial length of *L* = 198 Å is found for the *d*_001_ reflection (18 Å *d*-spacing). In other words, the 64MMT sample had a 001 polymer/clay (para)crystallite of *L* ∼200 Å, of which around 11 platelets were inside. This unexpected regularity at high particle content supports the findings of a closely packed lamellar structure.

**Table 1:** Equivalent Correlation (ξ) or Domain Length (*L*) and Number of Platelets (*n*) Estimated Using the 1D Scherrer Equation on 001 (from WAXS) and 003 (from Bragg-Bretano) Basal Reflections of Gelatin/MMT Composites^a^

	001					003				
sample	*q* (1/nm)	*a* (Å)	length (Å)	*n* (L/a)	Efron’s pseudo-*R*^2^ Gaussian	*q* (1/nm)	*a* (Å)	length (Å)	*n* (L/a)	Efron’s pseudo-*R*^2^ Gaussian
33MMT	0.017	37.5	**216.8**	**5.8**	0.996	0.051	12.2	**42.1**	**3.4**	0.935
44MMT	0.023	27.8	**156.7**	**5.6**	0.992	0.056	11.2	**50.6**	**4.5**	0.820
64MMT	0.035	18.2	**197.6**	**10.9**	0.990	0.107	5.9	**87.5**	**14.9**	0.970

a *q*: center of basal reflection; *a*: periodicity estimated from *q*; ξ or *L*: equivalent correlation or domain length; *n*: equivalent number of platelets within periodic length; and Efron’s pseudo-*R*^2^ gaussian: the Efron’s pseudo-*R*^2^ estimated for the Gaussian fit used to obtain full width at half maxima.

Altogether, the various gelatin/MMT compositions resulted in exfoliated structures of different lamellar spatial arrangements (regularity), as previously illustrated in Chart 1. We could identify exfoliated disordered (up to 33MMT), short-range ordered (up to 44MMT), and quasi long-range ordered at 64MMT (Figure 3b). Furthermore, the calculated equivalent number of platelets within periodic length (*n*) increased from the short-range to quasi long-range ordered composites–regardless of the scattering geometry (Table 1). This regularity is highly unexpected since water-based polymer composites reinforced with high aspect ratio nanoparticles can easily present long-range orientational order (high orientation factor or <*P*_*2*_> value) but seldom show a significant degree of positional order. In fact, to the best of our knowledge, positional order has been only reported in silicates modified by quaternary ammonium cations and host–guest additives.^27^

Our exfoliation strategy depends on the formation of a continuous network to immobilize the 2D material and develop yield stress. However, the extent of gelation or network density seemed to decrease with higher MMT loadings. The thermo-reversible gelation process is not a percolative one but it is caused by aggregation of helices into a collective fibrous network.^38^ In the XRDs, we observed the appearance of equatorial diffraction arcs up to 5MMT at *q* 0.06 nm^–1^, which were from the gelatin component (Figures 2 and 3). This nanoscale organization comes from the aggregation of renatured supramolecular helices. The crystallization was evidently hampered at elevated MMT content,^36,39,40^ above 11 vol %, in which additions also slowly increased the pH of the system closer to the protein isoelectric point. Clay negative interference on renaturation was also supported by differential scanning calorimetry (DSC) (Figure S8). This can be linked to the macroscopic breakdown of the gelatin-based physical network in the casting of gels (Figure S4). It is plausible that strong electrostatic and H-bonding interactions^40,41^ and, particularly, the decreased amount of loose chain ends disturbed the cooperative joining of helices. At elevated MMT fractions, the macroscopic gelatin cross-linking was heavily suppressed. We speculate that by introducing the clay, there is gelatin absorption on the clay surface, thereby preventing thermal gelation. Despite the formation of weaker gels, the network in these MMT samples was apparently sufficient to avoid clay stacking and phase separation.

Curiously, the fact that the samples at elevated filler content show quasi long-range order could be linked to a platelet-driven nanoconfinement of gelatin chains. As the system settles from water evaporation, the mobility of gelatin decorated MMT is reduced. The locked gelatin molecules will progressively get confined in the interlayers and lose the ability to form secondary structures. One could consider the typical length of gelatin random coil as a radius of gyration (*Rg*) in the order of 50 to 100 Å,^42^ coming from the rough estimate that *Rg* ∼ *aN*^1/2^ ∼ (10 Å)(100 units)^1/2^. This radius range is reasonably close to the increment in *d*-spacing at 58–64 vol % MMT, indicating protein confinement. We propose that the gelatin-specific binding is sufficient to prevent clay restacking and instead we observe colloid positional order caused by steric repulsion from the confined gelatin coils.

## 3 Conclusions

In conclusion, we hereby report on an easily attainable 2D material exfoliation strategy by implementing a hydrogel matrix with rapid network formation. If applied to solvent-based processes, this rationale can lead to controlled all-exfoliated systems for a wide loading range, as opposed to most reported results on nacre-like composites. For the thermo-reversible gelatin/MMT nanocomposites, we report filler loadings as high as 64% volume fraction. In addition, controlled hydrogelation becomes a suitable alternative to laborious processes such as multilayer deposition, in situ polymerization, external field alignment, etc. The high level of exfoliation and alignment of the clay platelets allows for precise tuning of the sample *d*-spacing through the hereby described volumetric rule. Nevertheless, the extent of gelation needs to provide enough particle immobilization during the drying phase. The locking mechanism needs to sustain time scales higher than that of filler relaxation. Hence, the strategy should be applied to other systems on a case-by-case basis. Another reason for this is that the system properties are still dependent on initial polymer-particle compatibility, polymer-penetration energy, and interfacial interactions. Remarkably, particle order–disorder transitions were found to develop from these nanostructures, which requires further attention. For instance, the composites 33MMT to 64MMT were found to form very ordered phases, with crystallite sizes about 200 Å long. Our future work shall explore the proposed hydrogelation strategy with regard to the platelet orientation mechanism and its effect on anisotropic properties.

## Supporting Information Available

Methods (gel suspensions and film fabrication, FIB-SEM, conditions for WAXS and Bragg–Brentano XRD, correlation or domain length calculations, and DSC on humid films); TGA; FIB-SEM image treatment and analysis; inverted vials gelatin/MMT gel suspensions; composite films; powder XRD of commercial gelatin A; integration of WAXS at higher *q* angles; DSC analysis of gelatin/MMT films at 0.33 water activity; and correlation or domain length for all composite fractions (PDF)
