# Supplementary material for: Systematic Study of the Nanostructures of Exfoliated Polymer Nanocomposites
Source: Macromolecules. 2023 Sep 14;56(18):7579–86. doi: 10.1021/acs.macromol.3c00575 (PMC10537450; doi:10.1021/acs.macromol.3c00575)
Supplement: Supplementary file 1 — ma3c00575_si_001.pdf [file ma3c00575_si_001.pdf]

Supporting information

Systematic study of the nanostructures of exfoliated polymer nanocomposites

Suellen Pereira Espíndola†, Jure Zlopasa‡, Stephen J. Picken†**

† Advanced Soft Matter, Department of Chemical Engineering, Faculty of Applied Sciences, Delft University of Technology, Van der Maasweg 9, 2629 HZ Delft, The Netherlands

‡ Environmental Biotechnology, Department of Biotechnology, Faculty of Applied Sciences, Delft University of Technology, Van der Maasweg 9, 2629 HZ Delft, The Netherlands

*E-mail: S.PereiraEspindola-1@tudelft.nl.

*E-mail: S.J.Picken@tudelft.nl.

Contents

Supporting Texts ... *3*

Supporting Text S1. Experimental Section ... **3**

1. Materials ... 3
2. Gel Suspensions and Film Casting ... 3
3. Focused Ion Beam Scanning Electron Microscopy (FIB-SEM) ... 4
4. Wide-Angle (WAXS) and Bragg-Brentano X-ray Scattering ... 5
5. Correlation or Domain Length ... 6
6. Differential scanning calorimetry (DSC) ... 7

Supporting Text S2. Thermogravimetric Analysis (TGA) ... **8**

Supporting Text S3. FIB-SEM Image Treatment and Particle Analysis ... **10**

1. Length of Na-MMT Particles ... 12

Supporting Text S4. Correlation length ... **13**

Supporting Figures and Tables ... *15*

Gels ... **15**

Films ... **16**

Gelatin PXRD ... **17**

WAXS 1D integration ... **18**

DSC ... **19**

References ... *20*

Supporting Texts

Supporting Text S1. Experimental Section

1. Materials

Gelatin from porcine skin (Type A, 78-80 mM free COOH/100 g protein, 50,000−100,000 Da, gel strength 300, relative density 1.3 g cm^{-3}) was obtained from Sigma Aldrich and used as received. Sodium montmorillonite (Na-MMT), CLOISITE-Na+, with D_{50} particle size <25 μm, basal spacing d001 of 11.7 Å, and density 2.86 g cm^{-3} was supplied by BYK Chemie GmbH, Germany, and used without further purification or surface treatment. The aspect ratio length over thickness) of the dispersed Na-MMT is typically reported as ranging from 10 to 1000 nm. All chemicals used were of analytical grade.

2. Gel Suspensions and Film Casting

A gelatin stock solution (3 w/v % solids content) was initially prepared by dissolving the powder in deionized water at 50 °C for 1 h using a magnetic stirrer. The pH of gelatin solution was 5.2 ± 0.1 at 50 °C, which is below its reported isoelectric point of 7 – 9.5. Na-MMT was mixed in deionized water under vigorous stirring for at least 24 h to achieve a 3 w/v % delaminated dispersion. The pH of Na-MMT suspension was 8.2 ± 0.1 at 50 °C.

Film-forming suspensions of protein/clay were prepared by carefully mixing (pre-calculated) wet ratios of the gelatin stock solution and Na-MMT suspension at 70 °C. This thermal treatment to the Na-MMT slurry was necessary to avoid lumps and re-agglomeration in the mixtures with protein due to temperature influence on its solubility.[1] All the Na-MMT was dispersed, without any remaining visual agglomerates. The protein/MMT suspensions were further mixed at 400 rpm for 2 hours at 70 °C to allow for gelatin-clay interaction. The

pH at 50 °C of the casting gel suspensions ranged from 5.4 up to 7.7, increasing in value with higher Na-MMT addition. The hot dispersion was carefully poured into a 9 x 2 cm polystyrene petri dish and dried at ambient conditions (20 °C, 50% RH) to form a thin film. The drying step lasted around 4 days and free-standing films were obtained by peeling off the new layer. Subsequently, the films were vacuum-dried at 40 °C for 1 day to remove excess humidity. The final thickness was designed to be in the range of 200 µm, what was confirmed using a digital micrometer at five random locations.

The study aimed at a wide range of filler loadings ranging from 1 to 80 wt. % Na-MMT, on the composite weight basis. In solid-state, this should be equivalent to up to 65 vol.% Na-MMT. Two replicates were performed for samples in region of interest, containing high clay content, where we aimed to achieve 30 and 60 vol. % MMT. The MMT content was later confirmed via thermal gravimetric analysis (TGA) (Supporting Text S2). Throughout this study, the composite clay content is expressed as volume percentage (vol. %). The samples are denoted as XMMT, where X is the volumetric MMT fraction percentage in the composite.

For the following characterization analyses, the films were additionally conditioned for a week in a desiccator containing silica gel at room temperature. This step was necessary to ensure there was absence of freely bound water, as the gelatin polymer has a hydrophilic nature.

3. Focused Ion Beam Scanning Electron Microscopy (FIB-SEM)

Focused ion beam scanning electron microscopy (FIB–SEM) experiments were performed in FEI Helios G4 CX microscope. A conductive thin layer of Au (~10 nm thickness) and a protective thin layer of W (0.3 µm thickness) were deposited on the cross-sectional surface of specimens using a sputter coater and gas injection system respectively. The focused beam

was operated at 30 kV and 2.5/0.24 nA used respectively to carve a trench and perform slicing up to a nominal depth of 10 μm. After correcting for the tilt angle between specimen and SEM detector, a series of micrographs were recorded of the polished cross-sections exposed, with a secondary electron detector operated at 10 kV. When possible, the average filler size and inclination of around 50 filler particles were identified and measured (Supporting Text S3). The technique allowed to investigate micro-arrangement of MMT particles within the gelatin matrix. Image enhancement was needed, i.e. solving for "curtaining" effect in the milling direction and resolution, which was possible with the *polishEM* software tool[2]. Measurements and analysis of the micrographs were performed by *Gwyddion* software.

4. Wide-Angle (WAXS) and Bragg-Brentano X-ray Scattering

Wide-angle X-ray scattering (WAXS) was used to determine the composite nanostructure. Transmission mode X-ray diffraction was performed using a Bruker AXS D8 Discover with a VÅNTEC 2D detector and using Cu Kα radiation (λ = 1.54184 Å) at 50 kV and 1 mA. A point collimator of 0.3 mm was used, and the sample−detector distance was 30 cm parallel (incident beam at a glancing angle) and perpendicular to the film surface. Additional measurements were done with the detector shifted by $2\theta = 11.5°$ in order to obtain 2D patterns at higher angles while avoiding background noise.

Experiments were also conducted with a Bruker D8 Advance diffractometer (2theta-theta scan, often called Bragg-Brentano or focusing geometry) with Co Kα source (λ = 1.7889 Å, 35 kV and 40 mA) with Lynxeye position sensitive detector. The measurement range on a motorized varied divergent slit was set from 4 to 50 degrees with step size of 0.02 mm. A measuring time of 0.1 second per step was employed. For basic interpretation and data

curation of the X-ray diffraction (XRD), Bruker software (DiffracSuite.EVA version 5.1, Bruker, USA) was used.

To normalize for the different X-ray sources (λ), the XRD data is shown over the scattering vector q = (4π/ λ) sin θ, where 2θ is the scattering angle. The layer d-spacing was calculated from Bragg's law.

5. Correlation or Domain Length

In general, information on the size of ordered domains in composites can be extracted from the line shape of X-ray peaks. If we consider crystals with a three-dimensional lattice, the probability to find the center of mass of a molecule at a position *r* with respect to a test molecule (r = 0) can be described by a distribution function *D*(*r*). True dimensional positional order results in a series of Dirac delta functions along the *r* values. However, for quasi long-range and short-range order systems, such as nanocomposites, this distribution displays an algebraic decay with distance r, e.g., $D(r) \propto r^{-\eta}$ or $D(r) \propto e^{-\frac{r}{\xi}}$. The Fourier transform of the exponential decay corresponds to a Lorentzian expression, which is one of the distribution functions commonly fitted to the radially integrated X-ray profiles.[3]

In our case, the nanoplatelets will show a high aspect ratio if exfoliated, what favors the development of 1D ordered systems upon drying. Hence, distribution functions also can be fitted to estimate a correlation or domain length (ξ or L) as a measure for the degree of positional order.[4] To calculate this length, we applied the Scherrer equation, which is originally derived for a 1D finite stack of lattice planes:

$$\xi \; or \; L = \frac{K\,\lambda}{FWHM\,\cos\theta} \tag{S1}$$

where ξ or L are the average correlation length or domain size, λ is the wavelength of X-ray source, FWHM is the peaks' full width at half maxima and θ the diffraction angle (radians), respectively. The empirical proportionality factor (K) was assumed to be 0.89. A Gaussian fit

was chosen to obtain the FWHM, since we observed quasi long-range order and possible convoluted instrumental broadening of the scattered image. The Gaussian statistical goodness-of-fit was estimated by the probabilistic parameter of Efron's pseudo-R^2. The equivalent number of platelets within the period was estimated from the relationship: length over 00*l* d-spacing.

6. Differential scanning calorimetry (DSC)

Differential scanning calorimetry (DSC) experiments were performed to characterize the thermal behavior of gelatin/MMT composites. First, snippets from the film samples were acclimated to a 33% relative humidity environment using $MgCl_2$ saturated salt solution. The water uptake capacity at the end of two weeks was measured gravimetrically. The DSC method consisted of heating the sample from 223 to 425 K, at rate of 3 K min^{-1}, on a PerkinElmer Pyris diamond instrument with two 1 g furnaces and calibrated with indium. Nitrogen gas was used to purge the thermal analyzer at 50 $mLmin^{-1}$. Stainless steel pans with o-ring seals were used for hermetically encapsulating the equilibrated samples (20 mg). An identical empty reference pan was used. The pans were sealed according to supplier instructions (PerkinElmer). Data visualization was carried out by Python, in which the y-axis refers to endothermic transitions. Each thermogram was analyzed for the melting (or denaturation) event.

Supporting Text S2. Thermogravimetric Analysis (TGA)

The composite samples were cut to snippets and analyzed using a thermogravimetric analyzer (TGA 8000, Perkin Elmer, USA) from 30 to 900 °C using a heating rate of 10 °C min^{-1}. The sample weight was in the range of 4 to 6 mg. Corundum crucibles were used and air as a purge gas at a flow rate of 20 mL min^{-1}. The method included isothermal steps at 105 and 900 °C for water removal and final weight equilibration, respectively.

In Figure S1, the thermal mass loss of gelatin, clay, and hybrids samples casted from 3 w/v % mixtures are shown. The first gelatin weight loss occurs at 270 °C, due to gelatin decomposition, and the second one around 500 °C region, due to combustion of residual organics. For the nanocomposite samples up to 5MMT, it can be clearly observed that the secondary onset thermal decomposition of the composites, around 530 to 610 °C, is higher than that of the neat protein. It can also be noted that the thermal rate of decomposition of hybrids is obviously reduced with clay concentration, specially at higher MMT loadings (> 11MMT). Thus, the well-dispersed clay inhibited the weight loss of gelatin, effectively acting as a barrier element, and because of its high thermal decomposition. Due to the usage of oxidative environment and negligible ash content of gelatin, the final residue represents the weight percentage of MMT. These concentrations were later converted into vol. % MMT, as presented in caption of Figure S1, ranging from 0.4 to 64%.

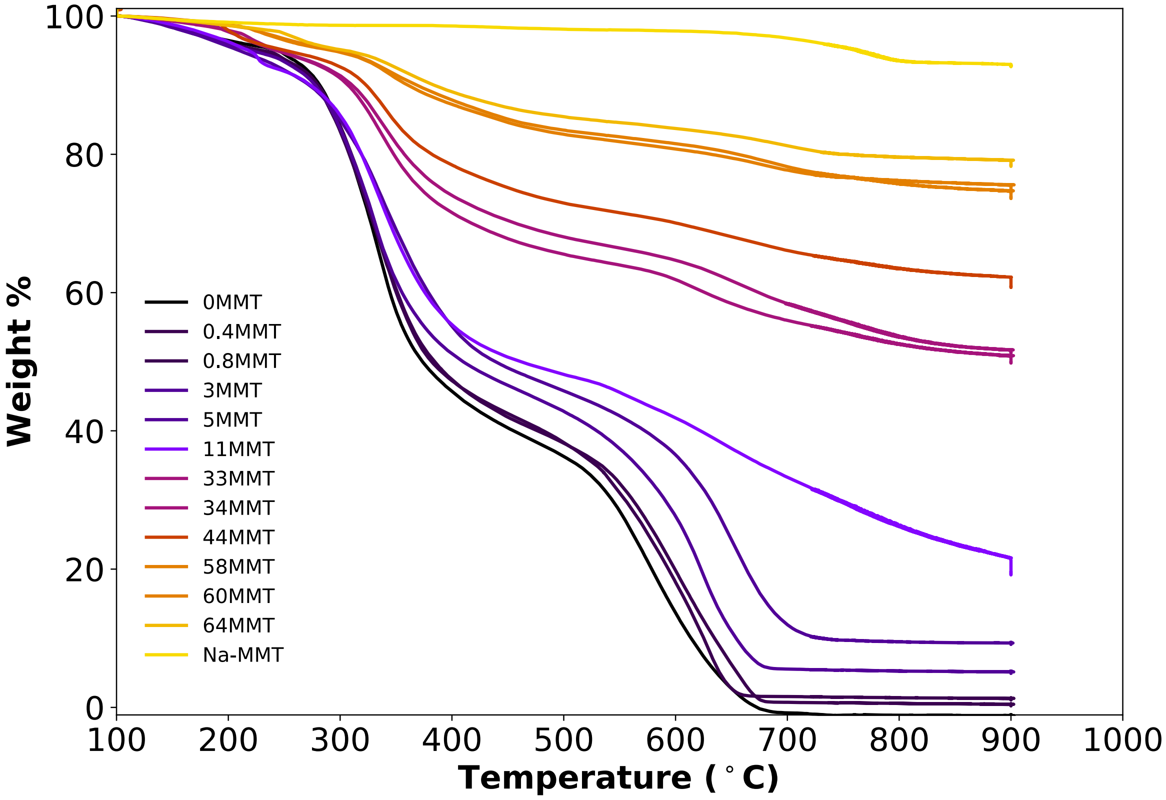

Figure S1. TGA of gelatin and gelatin/MMT composites fabricated from 3 w/v % suspensions at different filler loadings, shown in volume fraction,

Supporting Text S3. FIB-SEM Image Treatment and Particle Analysis

The FIB-SEM sample images were first treated to remove typical ion beam streak artifacts or "curtaining" (Figure S2). The curtaining in the ion milling direction depends on the sensitivity of the material, being common in composites of hard and soft materials, and arising from uneven milling. This image treatment was done via 2D Fast Fourier Transformation (*polishEM* software tool).

In the case of sample Gelatin/0.4MMT, due to the low loading of MMT clay and its electron density, it was possible to observe the internal particle dispersion and composite structure. The MMT nanoplatelets are possible to be visualized only due to the high electron density contrast between the organic matrix and silicate filler. Because of the same phenomena, the visible particles are actually a blurred image of the real nanosized structure, which can only be further resolved with X-ray analysis.

Unlike previous, the FIB-SEM image of sample 11MMT was too convolved, a result from high charging interference. However, a dense and layered structure was observed, which was also free of clay aggregate traces. At 11MMT, the mean layer spacing was measured roughly 76 to 181 nm. The nanoparticles cannot be observed individually already at this volumetric content (Figure S2b and c). At high volume fraction (64MMT), the layered sample shows undulation features but it is also predominantly free of stacking.

No gelatin orientation was observed in any of the studied samples. However, during ion milling temperatures can shortly reach the onset melting point of solid gelatin ($\geq$ 87 °C or 360 K)[5], what hampers the observation of possible gelatin layers from the crystallization of helices.

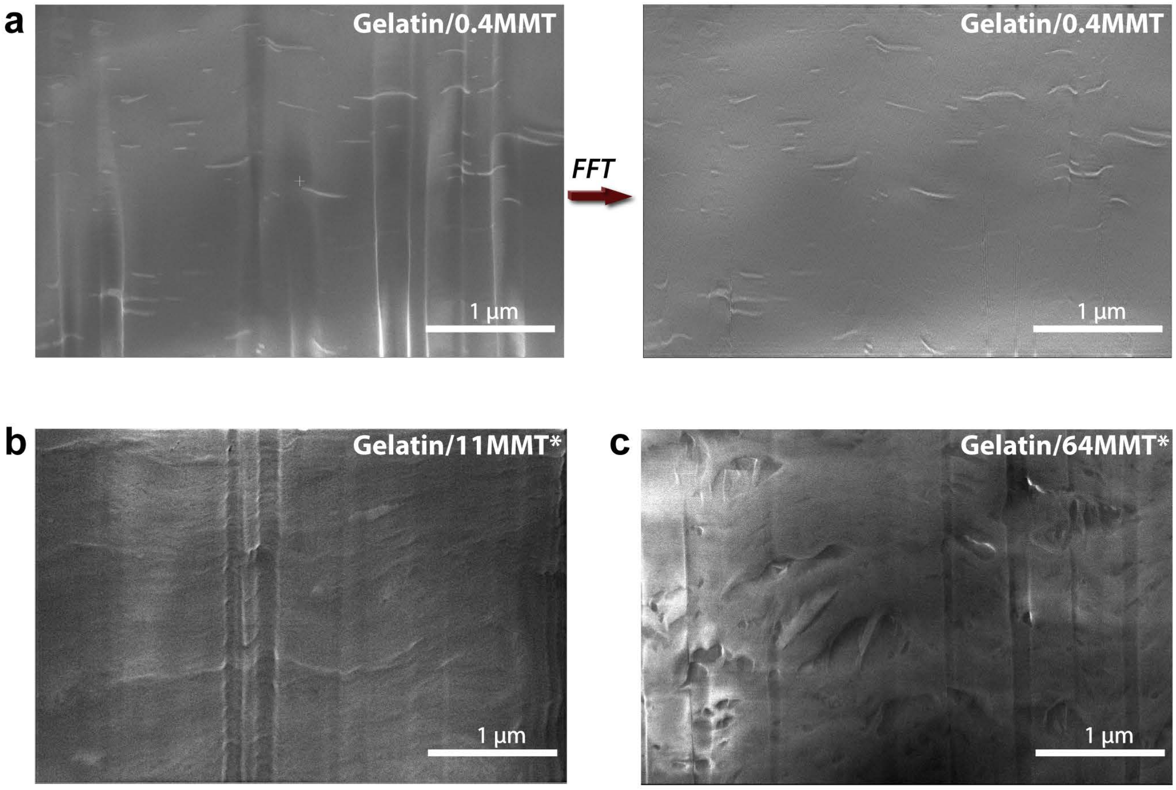

* nanoparticles cannot be resolved with FIB-SEM

Figure S2. FIB-SEM images of gelatin/MMT nanocomposites: (a) 0.4MMT unprocessed (left) and with curtaining removed via 2D FFT (right); (b) unprocessed 11MMT and (c) unprocessed 64MMT.

1. Length of Na-MMT Particles

The average particle length was estimated from three micrographic pictures belonging to the 0.4MMT sample (Figure S3a). Image analysis resulted in 212 ± 97 nm as average length of dispersed particle (Figure S3b). Furthermore, the (in-plane) inclination angle of particles coarsely tends to zero, suggesting sample anisotropy along this direction (Figure S3b and c).

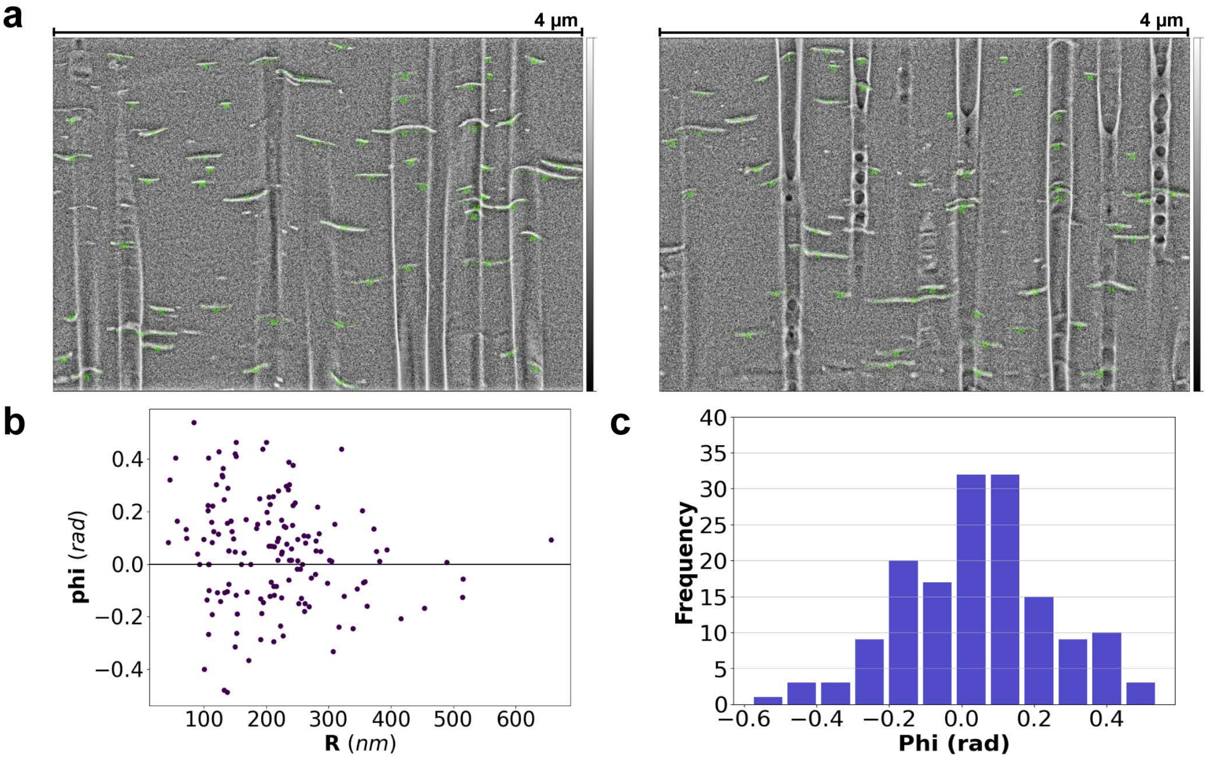

Figure S3. (a) FIB- SEM images of gelatin/0.4MMT showing imaging software particle identification and data. (b) R: total planar distances of particles, in nm; and Phi: inclination angle of particles, in radians. (c) The frequency distribution of inclination angle of MMT particles, Phi.

Supporting Text S4. Correlation length

Table S1 shows the equivalent correlation (ξ) or domain length (L) calculated from the 1D Scherrer equation on 001 (from WAXS) and 003 (from Bragg-Bretano) basal reflections for complete set of the gelatin/MMT composites. Since different scattering geometries were employed, some variation in correlation length between the [001] and [003] reflections should be expected. Thus, the correlation length should be evaluated in a relative sense. The width of [001] reflection measured via WAXS is the main reference supporting the crystallite size.

For the gelatin/MMT system, the samples are likely paracrystalline and do not have a "truncated" finite stack of platelets in resemblance to a single crystal. The definition of paracrystalline means that the peak width depends on the order of reflection. Therefore, the correlation length will vary (ξ or L) and the distribution of stacks in three-dimensional space becomes more complex. Moreover, even though we calculate that the correlation length is longer for [001] this needs further X-ray verification with using the same geometry and possibly synchrotron XRD.

In addition, it is relevant to note that the estimated number of platelets within the periodic length (n) was increasing and reasonably well-defined across the techniques, even with the loss of information. This indicates that the trend in increasing platelet stack "height" (n) was qualitatively in full agreement among the two experiments and further evidence of the order transition.

Table S1. Equivalent correlation (*ξ*) or domain length (*L*) and number of platelets (*n*) estimated using the 1D Scherrer equation on 001 (from WAXS) and 003 (from Bragg-Bretano) basal reflections of all gelatin/MMT composites.

Sample	001					003				
	q (1/nm)	*a* (Å)	Length (Å)	*n* (*L/a*)	Efron's pseudo-R^2 gaussian	*q* (1/nm)	*a* (Å)	Length (Å)	*n* (*L/a*)	Efron's pseudo-R^2 gaussian
33MMT	0.017	37.5	**216.8**	**5.8**	0.996	0.051	12.2	**42.1**	**3.4**	0.935
34MMT	0.018	34.6	**216.7**	**6.3**	0.999	0.054	11.7	**46.1**	**3.9**	0.941
44MMT	0.023	27.8	**156.7**	**5.6**	0.992	0.056	11.2	**50.6**	**4.5**	0.820
58MMT	0.033	18.9	**148.1**	**7.8**	0.998	n.a.	n.a.	**n.a.**	**n.a.**	n.a.
60MMT	0.035	18.1	**144.1**	**8.0**	0.960	n.a.	n.a.	**n.a.**	**n.a.**	n.a.
64MMT	0.035	18.2	**197.6**	**10.9**	0.990	0.107	5.9	**87.5**	**14.9**	0.970

q: center of basal reflection; *a*: periodicity estimated from *q*; *ξ* or *L*: equivalent correlation or domain length: *n*: equivalent number of platelets within periodic length; Efron's pseudo-R^2 gaussian: the Efron's pseudo-R^2 estimated for the Gaussian fit used to obtain full width at half maxima.

n.a. Experiment not available.

Supporting Figures and Tables

Gels

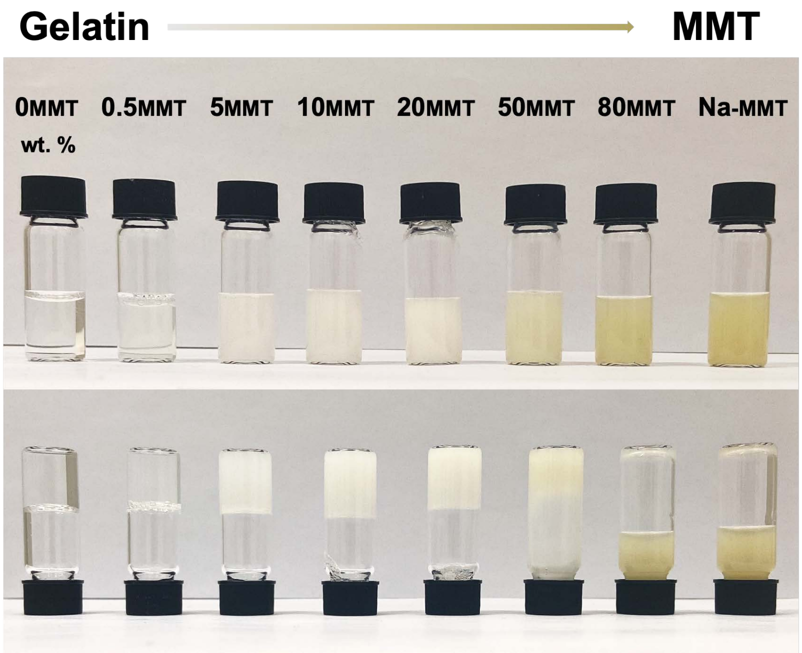

Figure S4. Vials containing gelatin/MMT film-forming suspensions (3 w/v % total solids). The notation XMMT classifies the suspension in terms of aimed MMT mass fraction (X%), on a gelatin/MMT basis. The inverted vials, at equilibrium, indicate that the hydrogel network starts to collapse at MMT loadings higher than 20 wt.%, which are gels resulting in composites with filler fractions above 11 vol. %.

Films

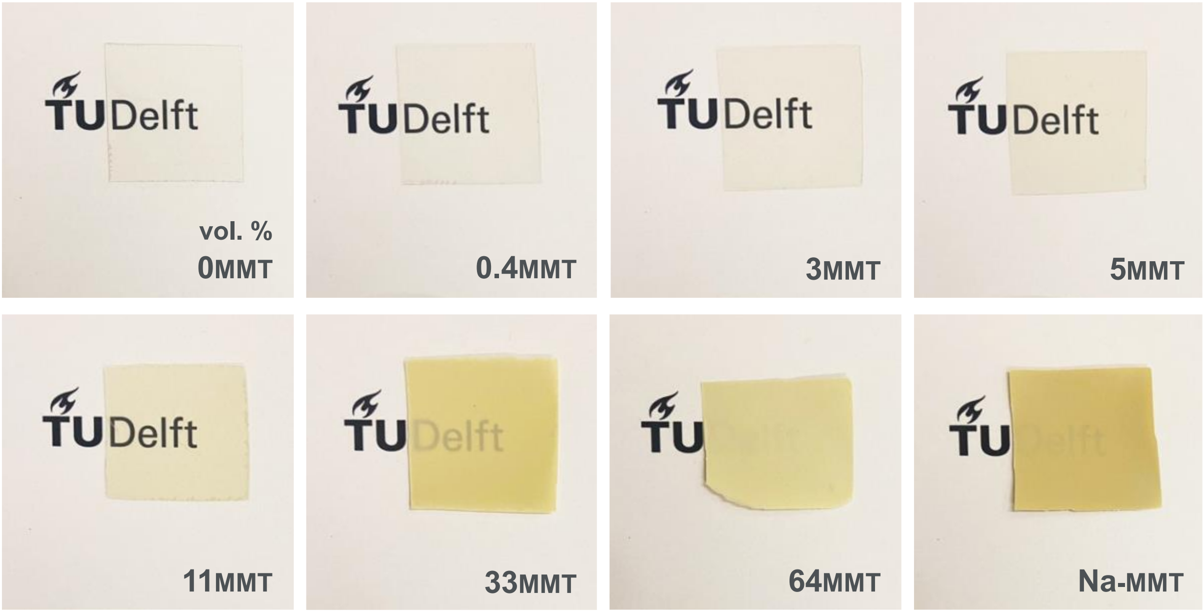

Figure S5. Images of gelatin/MMT nanocomposite films. The film thickness was around 0.2 mm. The notation XMMT classifies the composite film in terms of the determined MMT volume fraction (X%), on a gelatin/MMT basis.

Gelatin PXRD

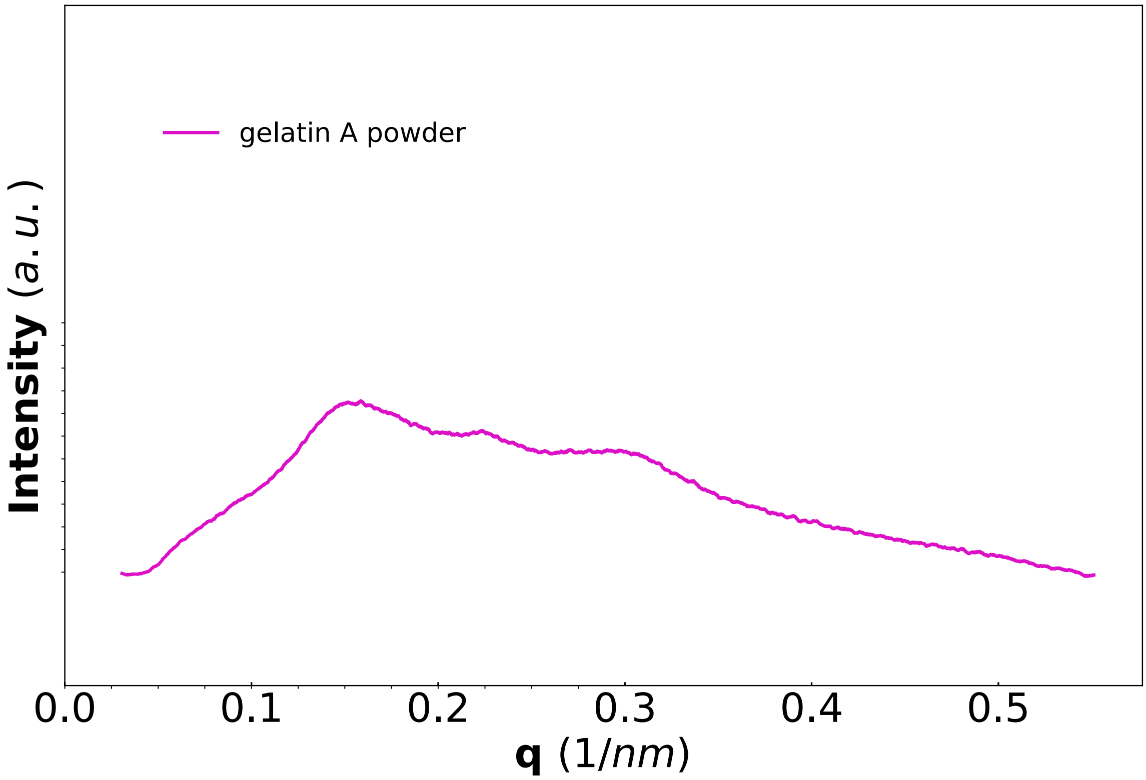

Figure S6. Powder XRD of the commercial gelatin type-A used in this study. The diffractogram shows that the *as is* gelatin is amorphous before processing into a solvent-casted film.

WAXS 1D integration

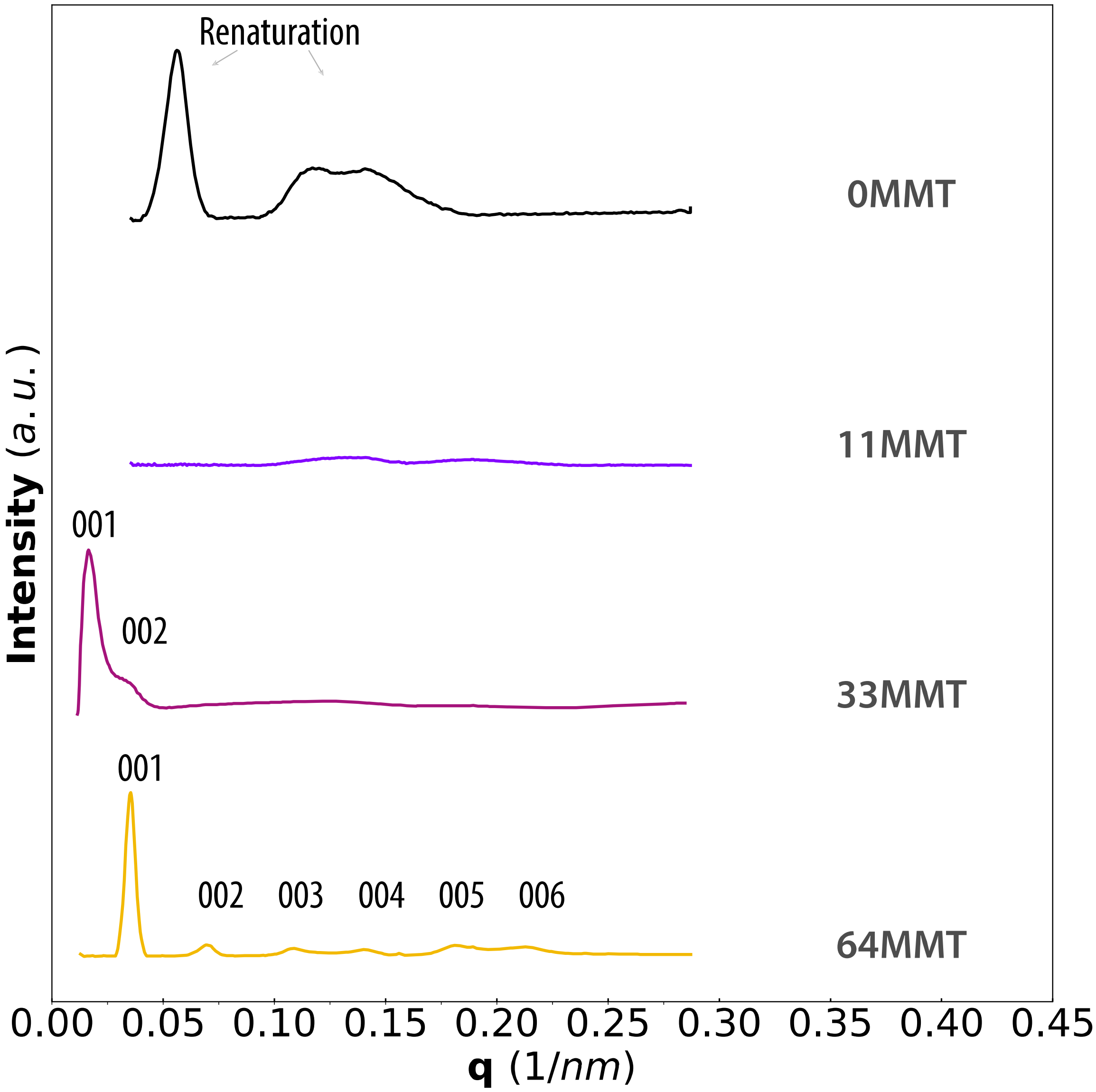

Figure S7. WAXS radial 1D integration of gelatin/MMT composites at higher q range and from a parallel glancing angle.

DSC

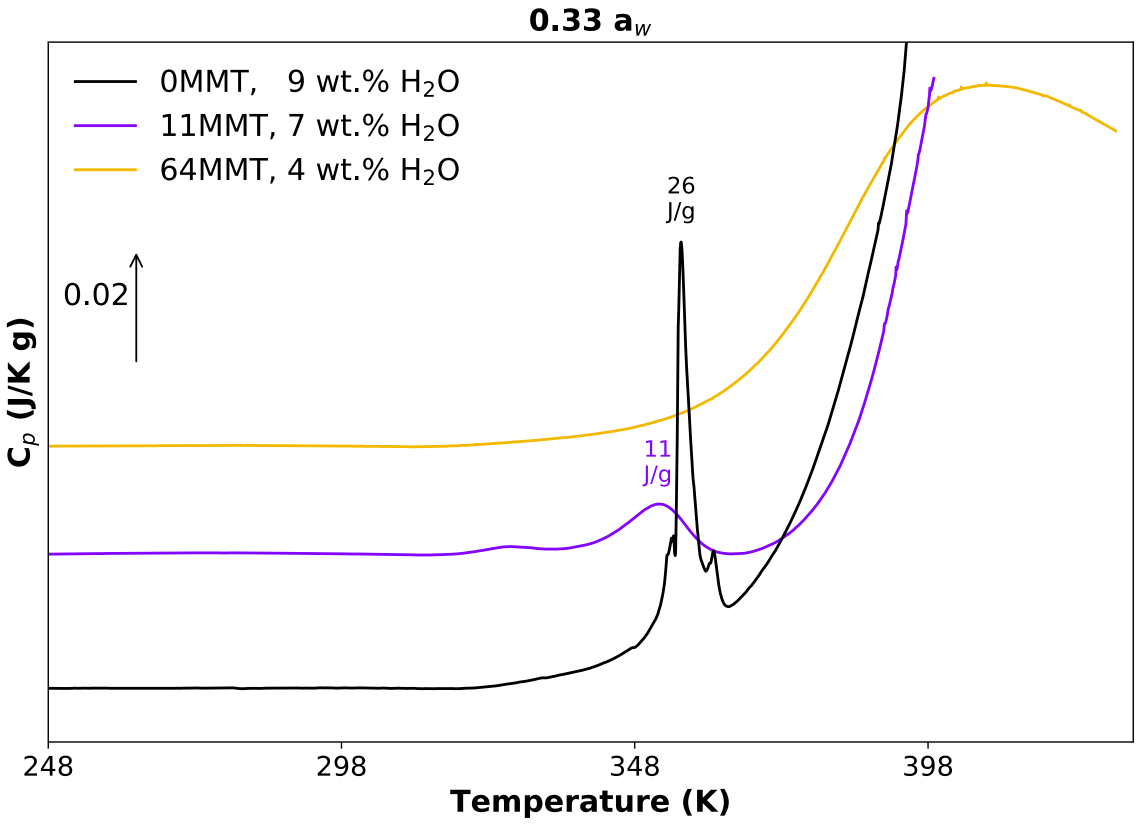

Figure S8. DSC analysis of the temperature dependance of heat capacity (endo up) of gelatin/MMT films equilibrated to a 0.33 water activity (a_w). The equilibrium water uptake for each sample was measured gravimetrically and is shown in legend. The blurred area assigned to the denaturation temperature T_{Hel} was suppressed with increasing clay content, as observed via absent or decreased corresponding energetic barrier (J/g) at transition. This suggests that more amorphous gelatin structures were formed with increasing clay volume fraction. Above 373 K, the DSC pan seal integrity is broken and we observe sample water loss.

References

(1) Zheng, J. P.; Xi, L. F.; Zhang, H. L.; Yao, K. D. Correlation between Reaction Environment and Intercalation Effect in the Synthesis of Gelatin/Montmorillonite Hybrid Nanocomposite. *J Mater Sci Lett* **2003**, *22* (17), 1179–1181. https://doi.org/10.1023/A:1025332029976.

(2) Fernandez, J. J.; Torres, T. E.; Martin-Solana, E.; Goya, G. F.; Fernandez-Fernandez, M. R. PolishEM: Image Enhancement in FIB-SEM. *Bioinformatics* **2020**. https://doi.org/10.1093/bioinformatics/btaa218.

(3) De Jeu, W. H. *Basic X-Ray Scattering for Soft Matter*; Oxford University Press, 2016.

(4) Yoshizawa, A.; Kato, Y.; Sasaki, H.; Takanishi, Y.; Yamamoto, J. Optically Isotropic Homochiral Structure Produced by Intercalation of Achiral Liquid Crystal Trimers. *J Phys Chem B* **2016**, *120* (21), 4843–4851. https://doi.org/10.1021/acs.jpcb.6b01242.

(5) Rao, Y. Q. Gelatin-Clay Nanocomposites of Improved Properties. *Polymer (Guildf)* **2007**, *48* (18), 5369–5375. https://doi.org/10.1016/j.polymer.2007.06.068.
